# A New Targeted Treatment for Lung Cancer Patients

**DOI:** 10.6004/jadpro.2012.3.2.5

**Published:** 2012-03-01

**Authors:** Beth Eaby-Sandy

**Affiliations:** From Abramson Cancer Center, Philadelphia, Pennsylvania

Review of "Anaplastic lymphoma kinase inhibition in non–small-cell lung cancer," by Kwak et al. (2010), New England Journal of Medicine, 363(18), 1693–1703. For a discussion of waterfall plots, both in general and how they are used in the Kwak et al. article, see the related article by Theresa Gillespie, PhD, MA, RN, on page 106.

Lung cancer is the number 1 cancer killer in the United States, resulting in more deaths than breast, colon, and prostate cancers combined per year (American Cancer Society, 2010). Non–small cell lung cancer (NSCLC) is the most common type of lung cancer, accounting for 85% of all lung cancer diagnoses. Lung cancer researchers have identified numerous targets for treatment. While many are still in clinical trials, crizotinib (Xalkori), which targets the *EML4-ALK* oncogenic fusion gene indentified in NSCLC, was recently approved by the US Food and Drug Administration (FDA).

Anaplastic lymphoma kinase (ALK) is a human gene that can also become an activating mutation found in several types of cancer. Echinoderm microtubule-associated protein-like 4 (*EML4*) is a gene protein also found in humans. In NSCLC, *EML4* and ALK are genes that can be translocated or rearranged and fused together, resulting in the *EML4-ALK* oncogenic fusion gene. The presence of the *EML4-ALK* fusion gene signals significant kinase activity, resulting in cancer cell proliferation and metastasis. *EML4-ALK* is identified using fluorescence in situ hybridization (FISH) on tumor pathology or cytology specimens. The Vysis *ALK* Break-Apart FISH Probe Kit is currently the only FDA-approved FISH test to detect *EML4-ALK*.

The *EML4-ALK* gene rearrangement in NSCLC is rare, present only in about 2% to 7% of all NSCLC patients; it is most prevalent in light or never-smokers. It is almost exclusive to adenocarcinoma histology and mutually exclusive in patients with epidermal growth factor receptor (EGFR) or KRAS mutations (Kwak, 2010). While this gene rearragement is uncommon, the test for *EML4-ALK* should be considered for all NSCLC patients with adenocarcinoma histology who have adequate tissue for sampling. In light or never-smokers with adenocarcinoma histology, a rebiopsy should strongly be considered if there is inadequate tissue available for testing.

## Important Crizotinib Trials

Data demonstrating the activity of crizotinib are reported from two clinical trials, a phase I trial (study 1001) and a phase II single-arm trial (PROFILE 1005). Both studies are still ongoing for measurement of outcomes. The first reported data came from the phase I study (1001), published in a New England Journal of Medicine article in 2010: an interim analysis of 82 *ALK*-positive lung cancer patients treated with crizotinib (Kwak, 2010). This study boasted a 57% overall response rate. Of the responses, there were 46 partial responses and 1 complete response. An additional 33% of patients had stable disease, totaling a 90% disease control rate. Median progression-free survival had not been met at the time of this publication because 63 out of the 82 patients were still on crizotinib treatment.

The phase II single-arm trial (PROFILE 1005) was presented in an abstract at the American Society of Clinical Oncology (ASCO) annual meeting in 2011, further expanding on data with crizotinib. Crino et al. (2011) provided data from 136 *EML4-ALK*-positive patients taking crizotinib, 76 of which were evaluable for tumor response. In this study, 93% of patients had received two or more chemotherapy regimens prior to receiving crizotinib. The results showed 83% of patients had tumor shrinkage, with 41 of the 76 patients having > 30% tumor shrinkage. Only 7 out of the 76 patients developed objective tumor progression.

## Adverse Events

Nausea has been the most commonly reported gastrointestinal adverse event, occurring in about 50% to 60% of patients; however, none experienced grades 3/4 nausea (Kwak, 2010; Crino, 2011). Other reported side effects can be seen in Table 1. One unique side effect to this drug not commonly seen in other targeted agents is the presence of visual disturbances. This side effect has been described as difficulty with visual accommodation when going from dark to light, with a trail of light following an object (Kwak, 2010). It occurs most commonly within 30 minutes of taking the drug and often abates the longer the patient is on treatment. Increases in liver function tests have been reported, with 6% being grades 3/4 (Kwak et al., 2010). There were nine deaths in PROFILE 1005 reported in the Crino (2011) study, two of which were considered treatment-related (pneumonitis and unknown cause).

**Table 1:**
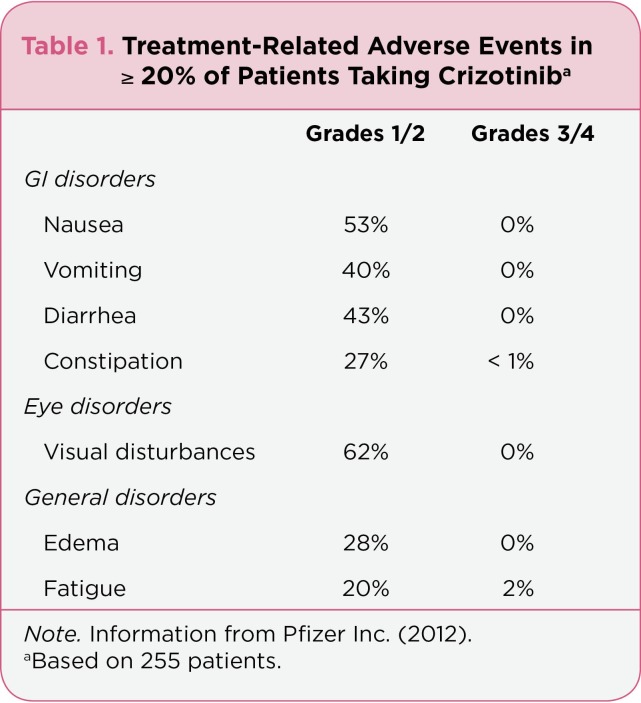
Table 1. Treatment-Related Adverse Events in = 20% of Patients Taking Crizotinib

## Response and Survival Data 

The impressive overall response rate, progression-free survival, and minimal toxicities have lead to the rapid approval of crizotinib. Overall survival data with crizotinib have not been determined at this time, but studies are ongoing to track and collect survival data. It has been studied mainly in the second-line or later treatment setting in *-ALK*–positive NSCLC. Only 13% of patients in the phase I trial (study 1001) were treatment naive, and 0 patients in the phase II trial (PROFILE 1005) were treatment naive.

Typical response rates for chemotherapy in the second-line setting of NSCLC have been inferior to the response rates seen in the crizotinib studies. Response rates for the three FDA second-line approved agents for NSCLC are 9.1% for pemetrexed (Alimta), 8.9% for erlotinib (Tarceva), and 8.8% for docetaxel (Hanna et al., 2004; Shepherd et al., 2005). To date, the overall response rates for crizotinib in the phase I and II studies were 57% and 83%, respectively.

## Future Directions

There is an ongoing randomized phase III clinical trial, PROFILE 1007, comparing crizotinib to standard chemotherapy in the second-line setting for ALK-positive NSCLC patients (Pfizer Inc., 2010). Further data on progression-free survival, overall survival, and clinical benefit will be reported when the data from the phase I and II trials mature and the phase III trial is completed.

## Implications for Advanced Practitioners

Crizotinib is a capsule taken orally at 250 mg twice a day. It is also commercially available in 200-mg tablets when dose reductions become necessary. The capsule should not be opened or dissolved, and it can be taken with or without food. Crizotinib should be continued twice a day until disease progression or development of toxicity that warrants suspending or discontinuing therapy.

Advanced practitioners (APs) are often disconnected with patients while they are taking oral cancer treatments. The patients are prescribed their medication, but they are sent home rather than spending time in the infusion suite where they receive reinforced teaching from oncology infusion nurses. Therefore, patients should be counseled on methods for oral adherence such as utilizing a pillbox or other reminders to take the drug on schedule.

Additionally, financial barriers such as high copays can play a major role in adherence and can be a source of high anxiety for patients. Advanced practitioners should work with the social worker to secure assistance when possible, either through the pharmaceutical company (e.g., co-pay assistance cards) or through charitable organizations. Patient follow-up with the AP in clinic shortly after initiating treatment with crizotinib is helpful to assess laboratory values, drug side effects, and adherence. Crizotinib offers high response rates to *-ALK*–positive NSCLC patients, and APs can play a very important role in maintaining these patients on therapy.

## Disclosure

Beth Eaby-Sandy has served on speakers bureaus for Merck & Co., Genentech, Inc., and Eli Lilly and Company, and has acted as a consultant for Amgen Inc.

## References

[1] American Cancer Society. (2010). Cancer facts & figures 2010. Atlanta, GA: American Cancer Society.. http://www.cancer.org/acs/groups/content/@epidemiologysurveilance/documents/document/acspc-026238.pdf.

[2] Crino L., Kim D., Riely P.A., Janne F. H., Blackhall D. R., Camidge V., Shaw A. T. (2011). Initial phase II results with crizotinib in advanced *ALK*-positive non-small cell lung cancer (NSCLC): PROFILE 1005 [Abstract 7514].. *Journal of Clinical Oncology*.

[3] Hanna N., Shepherd F. A., Fossella F. V., Pereira J. R., De Marinis F., von Pawel J., Bunn Jr. P. A. (2004). Randomized phase III trial of pemetrexed versus docetaxel in patients with non-small-cell lung cancer previously treated with chemotherapy.. *Journal of Clinical Oncology*.

[4] Kwak E. L., Bang Y-J., Camidge R., Shaw A. T., Solomon B., Maki R. G., Iafrate A. J. (2010). Anaplastic lymphoma kinase inhibition in non-small-cell lung cancer.. *New England Journal of Medicine*.

[5] Pfizer Inc. (2010). PROFILE 1007.. http://www.pfizer.com/files/news/asco/crizotinib_study_1007_backgrounder_2010.pdf.

[6] Pfizer Inc. (2012). *Xalkori package insert.*.

[A7] Shepherd F. A., Pereira J. R., Ciuleanu T., Tan E. H., Hirsh V., Thongprasert S., Seymour L. (2005). Erlotinib in previously treated non-small-cell lung cancer.. *New England Journal of Medicine*.

